# Cyanidin-3-Glucoside Suppresses Cytokine-Induced Inflammatory Response in Human Intestinal Cells: Comparison with 5-Aminosalicylic Acid

**DOI:** 10.1371/journal.pone.0073001

**Published:** 2013-09-06

**Authors:** Diana Serra, Joana Paixão, Carla Nunes, Teresa C. P. Dinis, Leonor M. Almeida

**Affiliations:** 1 Center for Neuroscience and Cell Biology, University of Coimbra, Coimbra, Portugal; 2 Faculty of Pharmacy, University of Coimbra, Coimbra, Portugal; Institut Pasteur de Lille, France

## Abstract

The potential use of polyphenols in the prevention and treatment of chronic inflammatory diseases has been extensively investigated although the mechanisms involved in cellular signaling need to be further elucidated. Cyanidin-3-glucoside is a typical anthocyanin of many pigmented fruits and vegetables widespread in the human diet. In the present study, the protection afforded by cyanidin-3-glucoside against cytokine-triggered inflammatory response was evaluated in the human intestinal HT-29 cell line, in comparison with 5-aminosalicylic acid, a well-established anti-inflammatory drug, used in inflammatory bowel disease. For this purpose, some key inflammatory mediators and inflammatory enzymes were examined. Our data showed that cyanidin-3-glucoside reduced cytokine-induced inflammation in intestinal cells, in terms of NO, PGE_2_ and IL-8 production and of iNOS and COX-2 expressions, at a much lower concentration than 5-aminosalicylic acid, suggesting a higher anti-inflammatory efficiency. Interestingly, cyanidin-3-glucoside and 5-aminosalicylic acid neither prevented IkB-α degradation nor the activation of NF-kB, but significantly reduced cytokine-induced levels of activated STAT1 accumulated in the cell nucleus. In addition, we established that phosphorylated p38 MAPK was not involved in the protective effect of cyanidin-3-glucoside or 5-aminosalicylic acid. Taking into account the high concentrations of dietary anthocyanins potentially reached in the gastrointestinal tract, cyanidin-3-glucoside may be envisaged as a promising nutraceutical giving complementary benefits in the context of inflammatory bowel disease.

## Introduction

Anthocyanins belong to the family of flavonoids and constitute the largest group of water soluble pigments in nature, responsible for the blue and purple colours of many fruits and vegetables, being consequently widespread in the human diet. Due to their relatively high consumption, the impact of anthocyanins on health promotion and disease prevention has been extensively investigated in the last decades [1]–[7].

Although there is some controversy regarding bioavailability of polyphenols [8], [9], they can reach concentrations up to several hundred micromolar in the gastrointestinal tract [10]. This is due in part to their abundance in the diet and also to poor intestinal absorption.

Recently, it was reported that dietary polyphenols can modulate intestinal inflammatory response, an important component of Inflammatory Bowel Disease (IBD) pathogenesis [10], [11]. IBD is a chronic and relapsing inflammatory disorder of gastrointestinal tract that includes Crohn's disease (CD) and Ulcerative Colitis (UC). In spite of its etiology remains unclear, it is believed that its occurrence is related to a genetic susceptibility of the patient to develop an exaggerated immune response to one or more promoting factors, probably commensal microorganisms present in the intestinal flora [12]. Consequently, an uncontrolled inflammation is triggered leading to tissue destruction. 5-Aminosalicylic acid (5-ASA) is a well-established drug used in adults, particularly in the treatment of mild to moderate active UC or to maintain remission periods of UC. It is known that, in most cases, 5-ASA is rapidly and extensively absorbed before reaching the colon [13]. Moreover, 5-ASA is not free of adverse effects, although it is usually well tolerated [14].

The beneficial effects of polyphenols, including anthocyanins, in humans were initially attributed to their antioxidant capacity in the prevention of diseases associated with oxidative stress, such as atherosclerosis and diabetes. Lately, some authors pointed out that other action mechanisms could be involved in the pharmacological activity of polyphenols, namely by interfering with essential signaling pathways and gene regulation [6], [7], [15], [16].

Abnormal up-regulation of nuclear factor kB (NF-kB) pathway has been observed in IBD patients and found closely related to the severity of intestinal inflammation [17]. Activation of NF-kB promotes the expression of many pro-inflammatory genes, such as those for iNOS and COX-2 [18]. However, beyond NF-kB, other transcription factors must be taken into account, such as the signal transducer and activator of transcription 1 (STAT1), whose expression and activation are heightened in IBD patients [19]. This factor also regulates the transcription of several inflammation-associated genes, including iNOS and COX-2 [20]. Furthermore, there are many kinase pathways involved in the regulation of inflammatory response upstream transcription factors, which may also be important to unveil the mechanisms underlying the anti-inflammatory effects of polyphenols, namely p38 MAPK pathway. Actually, it has been reported that the activity of p38 MAPK is increased in patients suffering from IBD [21].

**Figure 1:**
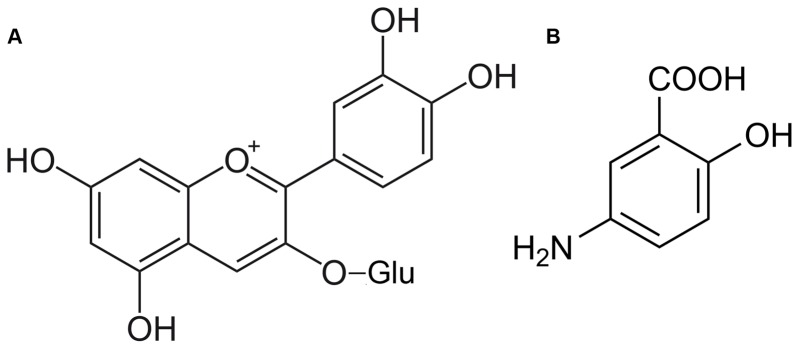
Chemical structures of cyanidin-3-glucoside (A) and 5-aminosalicylic acid (B).

Since increasing evidences support the efficacy of anthocyanins in modulating inflammatory response [5], [11], in the present study we attempted to scrutinize the mechanisms underlying cell signaling modulation induced by a typical dietary anthocyanin, in particular cyanidin-3-glucoside (C3G) -Figure 1A- which is one of the most abundant anthocyanins in nature, in the presence of an inflammatory stimulus. Thus, our main goal was to assess the protection afforded by C3G against cytokine-triggered inflammatory response in the human intestinal HT-29 cell line, used as an intestinal cell model, exploring its ability to counteract the expression of crucial pro-inflammatory enzymes and pro-inflammatory mediators, in comparison with 5-ASA (Figure 1B).

Our data evidenced that cell pre-incubation with 25 µM C3G or 500 µM 5-ASA was effective in down-regulating the production of NO, PGE_2_ and IL-8 and the expression of iNOS and COX-2 in cytokine-stimulated HT-29 cells. Interestingly, none of the compounds affected NF-kB activity. Conversely, they significantly inhibited STAT1 activation by modulating its phosphorylation. Since C3G was used in a much lower concentration than 5-ASA, C3G revealed a higher anti-inflammatory efficiency.

## Materials and Methods

### Reagents

Cyanidin-3-*o*-β-glucoside purified from natural sources was obtained from Extrasynthése (Genay, France). It had purity above 97% as measured by HPLC and was used as a solution in DMSO (5 mM) and stored in the dark, under nitrogen atmosphere, at −80°C.

Laboratory chemicals namely dimethylsulfoxide (DMSO), sodium dodecyl sulfate (SDS), 2,3-diaminonaphthalene (DAN), 3-(4,5-dimethylthiazol-2yl)2,5-diphenyl-tetrazolium bromide (MTT), phenylmethylsulfonyl fluoride (PMSF), streptomycin/penicillin, protease inhibitor cocktail and phosphatase inhibitors were purchased from Sigma-Aldrich Co.

For cell culture, Dulbecco's modified Eagle's medium (DMEM), 0.25% trypsin, fetal bovine serum (FBS) and phosphate-buffered saline (PBS) pH 7.4, were obtained from Gibco-Invitrogen.

Rabbit polyclonal antibody to iNOS and goat polyclonal antibody to phospho-STAT1 (Tyr701) were purchased from Santa Cruz Biotechnology (Santa Cruz, CA, USA); rabbit polyclonal antibody to COX-2 was purchased from Abcam (Cambridge, UK); rabbit polyclonal antibody to IκB-α and rabbit monoclonal antibody to phospho-p38 MAPK (Thr180/Tyr182) were purchased from Cell Signaling Technology (MA, USA); mouse monoclonal antibody to β-actin was purchased from Sigma-Aldrich Co and anti-rabbit, anti-mouse and anti-goat IgG secondary antibodies were obtained from Abcam (Cambridge, UK).

IL-1α, TNF-α and IFN-γ were purchased from Invitrogen (NY, USA).

### Cell culture

Human colon cancer cell line (HT-29) was obtained from European Collection of Cell Cultures (Porton Down, Salisbury, UK). Cells were grown in DMEM supplemented with 10% FBS, 100 U/ml penicillin and 100 µg/ml streptomycin at 37°C in a humidified atmosphere of 5% CO_2_. Cells were sub-cultured at confluence and used between the fourth and the twentieth passage. Before each experiment, cells at 80% confluence were starved in serum-free medium for 24 hours. Growth-arrested cultures, in medium without FBS, were treated according to the various experimental purposes.

HT-29 cells were stimulated with a cocktail of cytokines consisting of 10 ng/ml IL-1α, 20 ng/ml TNF-α and 60 ng/ml IFN-γ. Each cytokine was previously diluted in PBS with 1% BSA and then added to cells when convenient. Cells were pre-treated with C3G, 5-ASA or both for 1 hour before exposure to the cytokines and then maintained with the inflammatory stimulus for different time intervals, depending on the assay.

### Cell Viability

Cell viability was assessed by the mitochondrial-dependent reduction of 3-(4,5-dimethylthiazol-2yl) 2,5-diphenyltetrazolium bromide (MTT) to formazan, which is directly proportional to the number of living cells. After incubation for 24 hours with C3G and/or 5-ASA, 0.8×10^6^ cells/well in 6-well plates were washed with PBS and incubated with MTT (0.5 mg/ml) for 1 hour, at 37°C. Then, the medium was removed and the formazan crystals were dissolved in DMSO (900 µl). The extent of formazan formation was recorded at 530 nm in a Synergy HT plate reader.

Results were expressed as a percentage of control cells, *i.e.* non-treated cells.

### Measurement of Nitric Oxide Production

Nitric oxide production, in intestinal cells, was determined by measuring the amount of nitrite accumulated in cell culture supernatants. Nitrite was measured using a sensitive fluorimetric assay based upon the reaction of nitrite with 2,3-diaminonaphthalene (DAN), under acidic conditions, to form the fluorescent product 1-(H)-naphthotriazole [22]. Briefly, at the end of the incubation times, the supernatants were collected and nitrite was evaluated by adding 200 μl of freshly prepared DAN (0.025 mg/ml in 0.62 M HCl) to 200 μl of supernatant and mixed immediately. After 10 minutes incubation at room temperature in the dark, the reaction was stopped with 100 μl of 3 M NaOH. A standard curve was produced with known concentrations of sodium nitrite. Fluorescence intensity was read in a dual wavelength spectrophotofluorimeter, with excitation and emission at 365 nm and 405 nm, respectively. The sensitivity of the assay is 10 nM.

### Assessment of Prostaglandin E_2_ and IL-8 Production

Confluent HT-29 cells grown on six-well plates (0.8×10^6^ cells/well) were treated as above. After 16 hours of incubation, supernatants were collected and processed for PGE_2_ and IL-8 quantification, by using a competitive immunoassay kit (PGE_2_ EIA Kit) from Enzo Life Science and an Elisa Kit from RayBiotech, Inc, respectively, according to the manufacturer's instructions. The values were reported to protein content as measured by the Bradford assay (Bio-Rad, USA).

### Western-blot Analysis

Total, cytoplasmic and nuclear cellular protein extracts from several experiments were prepared and analyzed by Western-blotting. For total cellular protein extracts, washed cell pellets were resuspended in an ice-cold lysis buffer (50 mM Hepes pH 7.4, 150 mM NaCl, 2 mM EDTA, 10% (w/v) glycerol, 0.5% (w/v) sodium deoxycholate, 1% (v/v) Triton X-100, 1 mM PMSF, 1/100 (v/v) protease inhibitor cocktail) for 20 minutes, on ice. Cell debris was subsequently removed by centrifugation at 14000 rpm for 20 minutes at 4°C and supernatants were then collected and stored at −20°C. Cytoplasmic protein extracts were collected essentially in the same way. Washed cells were lysed in an ice-cold buffer containing 10 mM Tris–HCl, 10 mM NaCl, 3 mM MgCl_2_, 0.5% Nonidet P-40 and 1% protease inhibitor cocktail, pH 7.5, for 5 minutes on ice. Afterwards, lysates were centrifuged at 5000 rpm for 5 minutes at 4°C and the supernatants (cytoplasmic extracts) were collected and stored at −20°C. For nuclear cellular protein extracts, the pellets were collected and resuspended in an ice-cold buffer with 20 mM Hepes, 5 mM MgCl_2_, 0.2 mM EDTA, 1 mM DTT, 300 mM NaCl, 20% (w/v) glycerol, and 1% protease inhibitor cocktail, pH 7.5 and left on ice for 30 minutes. Then, lysates were centrifuged at 14000 rpm for 20 minutes at 4°C and the supernatants (nuclear extracts) were saved at −80°C.

Protein concentration was determined by using the Bio-Rad protein assay reagent (Bradford assay), according to the manufacturer's specifications (Bio-Rad, USA).

A range of 30–80 micrograms of reduced and denatured proteins were separated by SDS/PAGE electrophoresis on a 10%−12% (v/v) acrylamide gel and transferred onto polyvinylidene difluoride (PVDF) membranes (Amersham Biosciences, UK) by electroblotting. To avoid non-specific binding, membranes were blocked with skimmed milk in TBS buffer supplemented with 0.1% (v/v) Tween 20 (TBS-T: 20 mM Tris–HCl, 150 mM NaCl, 0.1% Tween 20) and then probed with antibodies against iNOS, COX-2 and IκB-α, overnight at 4°C and against phospho-STAT1 and phospho-p38 MAPK, 3 hours at room temperature, with a constant low shaking. Membranes were washed three times and then incubated with alkaline phosphatase-conjugated secondary antibodies (2 hours, room temperature, constant shaking). Immunoreactive complexes were detected by fluorescence in a Typhoon 9000 scanner (Amersham Biosciences). β-Actin was used as control for protein loading. Bands were analyzed using the ImageQuant TM software from Amersham Biosciences.

### Evaluation of NF-kB (p65) Activity

DNA-binding activity of NF-kB-p65 was measured in nuclear extracts using the TransAM^TM^ NF-kB-p65 protein assay (Active Motif, CA, USA), an ELISA-based method with high sensitivity and reproducibility.

For preparation of nuclear extracts, washed cells were lysed in an ice-cold buffer containing 10 mM Tris–HCl, 10 mM NaCl, 3 mM MgCl_2_, 0.5% Nonidet P-40 and 1% protease inhibitor cocktail, pH 7.5, for 5 minutes on ice. Afterwards, lysates were centrifuged at 5000 rpm for 5 minutes at 4°C and the supernatants (cytoplasmic extracts) were collected and stored at −20°C. The pellets were collected and resuspended in 50 µl of Complete Lysis Buffer (a solution provided by Active Motif, CA) and left on ice for 30 minutes. Then, lysates were centrifuged at 14000 rpm for 20 minutes at 4°C and the supernatants (nuclear extracts) were saved at −80°C.

DNA binding activity of p65 was evaluated with 15 µg of nuclear protein, according to the manufacturer's protocol and the results expressed in relative terms.

### Statistical Analysis

All data were expressed as means ± SEM of at least 3 independent assays, each one in duplicate. Differences between groups were analyzed by one-way analysis of variance (ANOVA), Tukey's was used as appropriate. Values of *p*<0.05 were accepted as statistically significant.

## Results

### 1. C3G and/or 5-ASA did not affect cell viability of HT-29 cells

**Figure 2:**
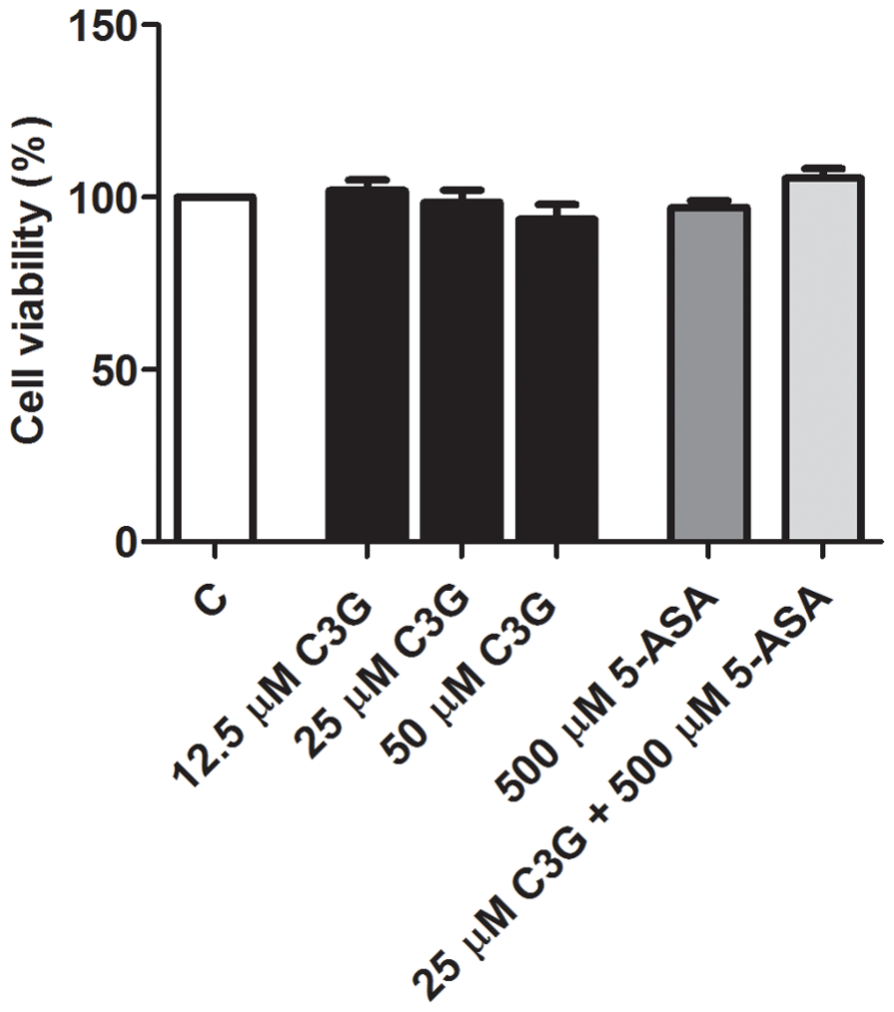
Effects of C3G and 5-ASA on the cell viability of HT-29 cells. HT-29 cells were incubated for 24 hours with different concentrations of C3G (12.5 to 50 µM), 5-ASA (500 µM) and a combination of 25 µM C3G and 500 µM 5-ASA. Cell viability was assessed by MTT reduction and determined as percentage of control cells (without compounds). Values are mean ± SEM of at least three different experiments, in duplicate.

In order to assess the cytotoxic effect of C3G and 5-ASA, a MTT assay was performed upon 24 hours of cell incubation with the compounds. As illustrated in Figure 2, neither C3G, in the concentration range of 12.5 to 50 µM, nor 500 µM 5-ASA alone, or in combination with 25 µM C3G, affected the percentage of cell viability relative to the control (cells without the compounds). In contrast, the cytokines, at the concentrations used as a cell stimulus, induced a decrease of cell viability to about 50 per cent (data not shown).

In the present study, both C3G (25 µM) and 5-ASA (500 µM) were used at subtoxic concentrations.

### 2. C3G inhibited secretion of pro-inflammatory mediators induced by the cytokines more efficiently than 5-ASA, in HT-29 cells

In order to evaluate the ability of C3G to inhibit pro-inflammatory mediators production, the levels of NO, PGE_2_ and IL-8 generated by cytokine-stimulated HT-29 cells were monitored.

**Figure 3:**
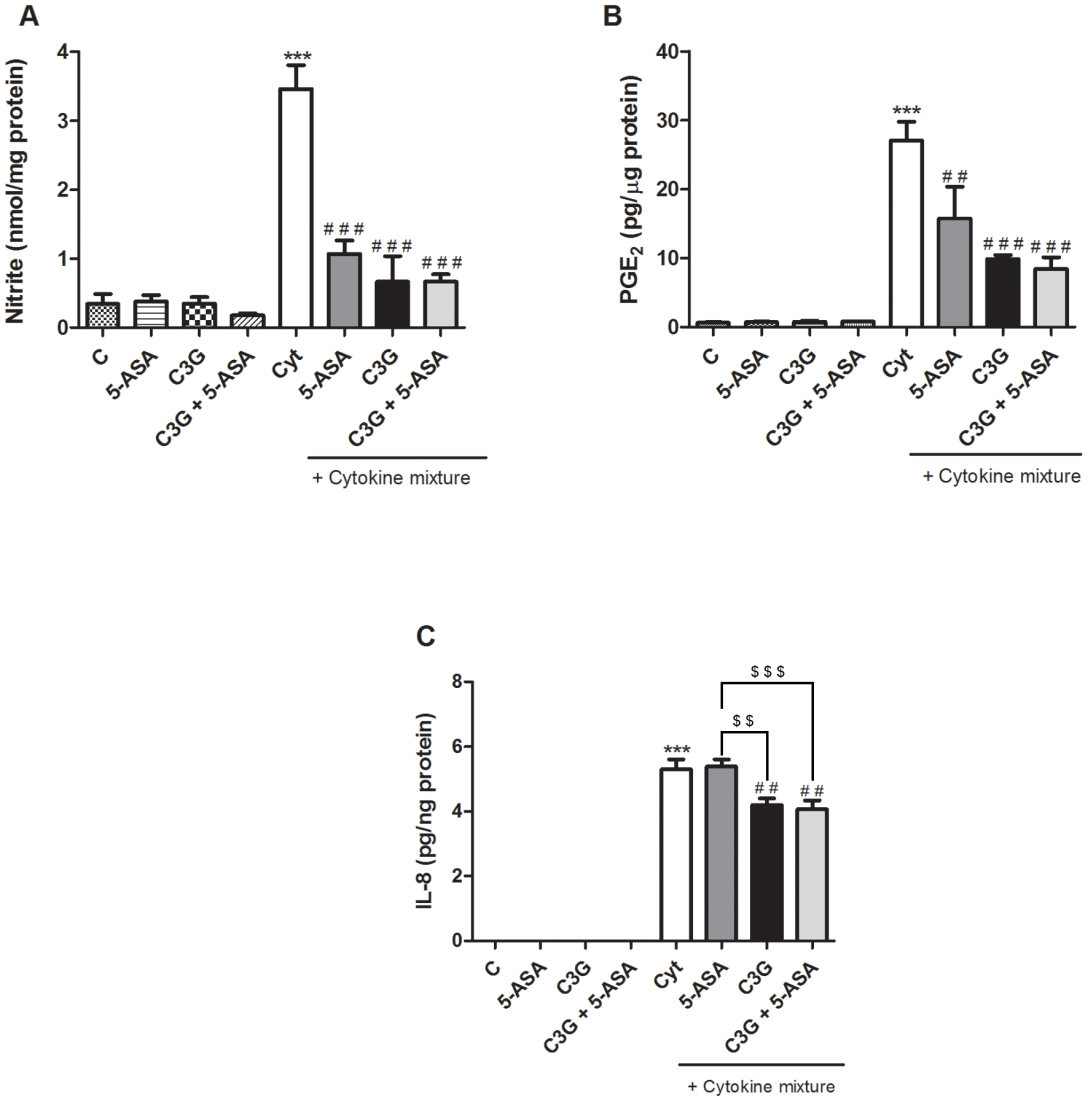
C3G and 5-ASA inhibit the production of pro-inflammatory mediators induced by cytokines in HT-29 cells. Cells were pre-incubated with 25 µM C3G or 500 µM 5-ASA or both (25 µM C3G plus 500 µM 5-ASA) and then treated with cytokines for a certain period of time. NO (A), PGE_2_ (B) and IL-8 (C) production by cells were measured as described in “Materials and Methods”. Values are mean ± SEM of at least three different experiments, in duplicate. ^***^ *P*<0.001 vs Control, ^##^ *P*<0.01, ^###^ *P*<0.001 vs Cytokines. ^$$^ *P*<0.01, ^$$$^ *P*<0.001 vs 5-ASA plus Cytokine mixture.

As shown in Figure 3A, stimulation of HT-29 cells with cytokines, for 24 hours, induced a strong cellular nitrite formation as compared to basal values found in non-stimulated cells. Treatment with 25 µM C3G, 500 µM 5-ASA or both, for 1 hour, before cytokine stimulation, significantly reduced the nitrite levels by about 75%. Although 25 µM C3G seems to be more efficient than 500 µM 5-ASA, the difference was not significant. Combination of the two compounds (25 µM C3G and 500 µM 5-ASA) caused no further effect. Previous studies of time-dependent release of NO demonstrated that until 16 hours of incubation there was no significant cellular nitrite formation.

To examine whether C3G, 5-ASA, or the combination of both, inhibited PGE_2_ and IL-8 production, cells were treated with/without the compounds for 1 hour and then treated with the cytokine mixture (IL-1α, TNF-α and IFN-γ) for 16 hours. In Figure 3B, it is clear that PGE_2_ production was enhanced in response to cytokine treatment and that this increase was strongly inhibited by C3G by almost 65%, a higher inhibitory effect than that induced by 5-ASA (about 50%). However, no additional significant effect was observed by the combination of C3G and 5-ASA.

Likewise, IL-8 production was deeply increased by the cytokines, but only C3G was able to significantly inhibit this production. In fact, as evidenced in Figure 3C, the presence of 5-ASA did not suppress the IL-8 production, contrary to C3G, which led to a decrease in its production by about 20%. The combination of C3G with 5-ASA did not improve the suppressive effect, which was similar to that of C3G alone.

### 3. C3G, like 5-ASA, inhibited cytokine-induced up-expression of iNOS and COX-2 in HT-29 cells

**Figure 4:**
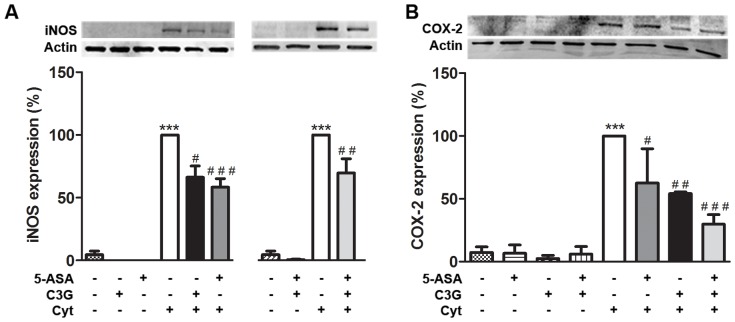
C3G inhibits cytokines-induced up-expression of iNOS and COX-2, like 5-ASA, in HT-29 cells. Cells were pre-incubated with 25 µM C3G or 500 µM 5-ASA or both (25 µM C3G plus 500 µM 5-ASA) and then treated with a combination of cytokines. iNOS (A) and COX-2 (B) expressions were evaluated after 24 hours or 16 hours, respectively, in total extracts by Western blotting, as described in “Materials and Methods”, and expressed as percentage of control. Values are mean ± SEM of at least three different experiments, in duplicate. ^***^ *P*<0.001 vs Control, ^#^ *P*<0.05,^ ##^ *P*<0.01, ^###^ *P*<0.001 vs Cytokines.

In order to assess whether the C3G or 5-ASA-induced decrease in the pro-inflammatory mediators levels, observed into the cell culture media, was exerted via inhibition of the inducible forms of NO synthase and cyclooxygenase, protein expressions of these enzymes were determined by Western-blotting. As shown in Figure 4, in non-stimulated cells, the expression levels of iNOS (Figure 4A) and COX-2 (Figure 4B) were very low or undetectable. However, in response to cytokine stimulation and after 24 hours or 16 hours, the levels of iNOS and COX-2, respectively, were up-regulated. When cells were pre-treated with the compounds in study their expression was significantly reduced. Worth of notice is the difference in the concentrations used in this work, *i.e.*, 25 µM C3G and 500 µM 5-ASA. These concentrations were previously tested by some of us, in other studies, either with anthocyanins or with 5-ASA [6], [23]. Thus, as shown in Figure 4A the inhibitory effect of C3G is slightly smaller than that of 5-ASA, this is not relevant bearing into consideration the highest concentration of 5-ASA (20 times).

In regarding to COX-2 expression (Figure 4B), C3G more efficiently down-regulated COX-2 expression than 5-ASA and the combination of C3G and 5-ASA afforded a much better protection than the individual compounds.

### 4. C3G and/or 5-ASA did not inhibit cytokine-induced NF-kB activation in HT-29 cells

**Figure 5:**
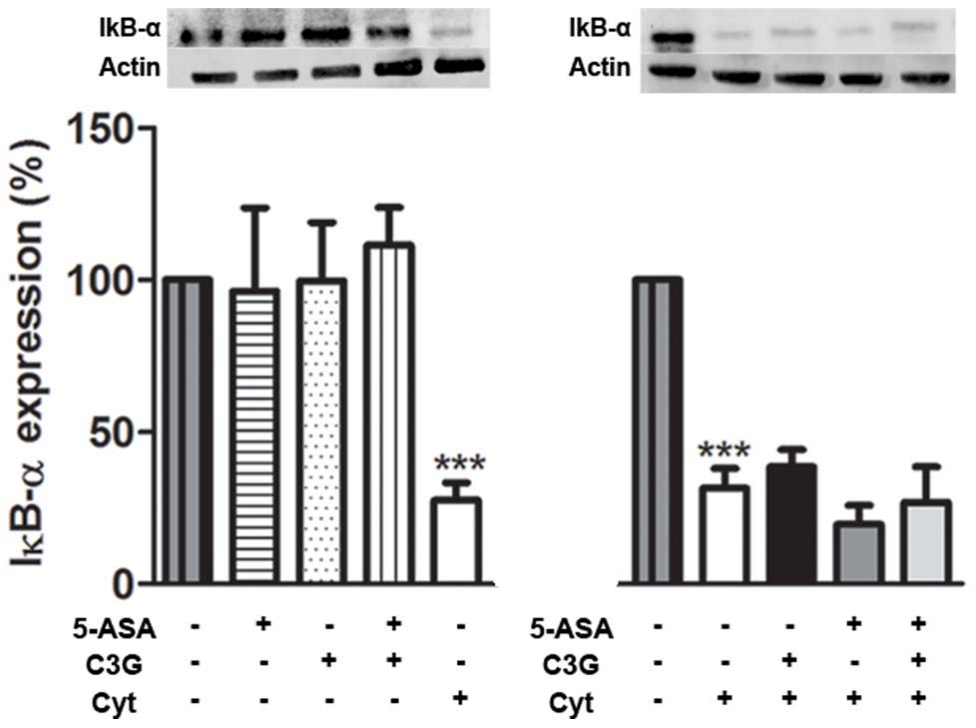
Neither C3G nor 5-ASA prevent the cytokine-induced IkB-α degradation in HT-29 cells. Cells were pre-incubated with 25 µM C3G or 500 µM 5-ASA or both and then treated with a combination of cytokines for 30 minutes. IkB-α degradation was evaluated in cytoplasmic extracts by Western blotting, as described in “Materials and Methods”. Values are mean ± SEM of at least three different experiments, in duplicate. ^***^ *P*<0.001 vs Control.

In an attempt to clarify the biochemical mechanism underlying the activation of iNOS, COX-2 and IL-8 observed in cytokine-stimulated HT-29 and the corresponding protection in pre-treated cells with C3G and/or 5-ASA, we evaluated the putative effects on NF-kB activation. As shown in Figure 5, cells stimulation for 30 minutes led to a decrease of cytoplasmic IkB-α by about 70% of the control, *i. e.* non stimulated cells. Some decrease was observed as soon as 15 minutes (data not shown), but more intensely at 30 minutes. However, cells pre-incubation with C3G and/or 5-ASA did not hamper cytokine-induced IkB-α degradation.

**Figure 6:**
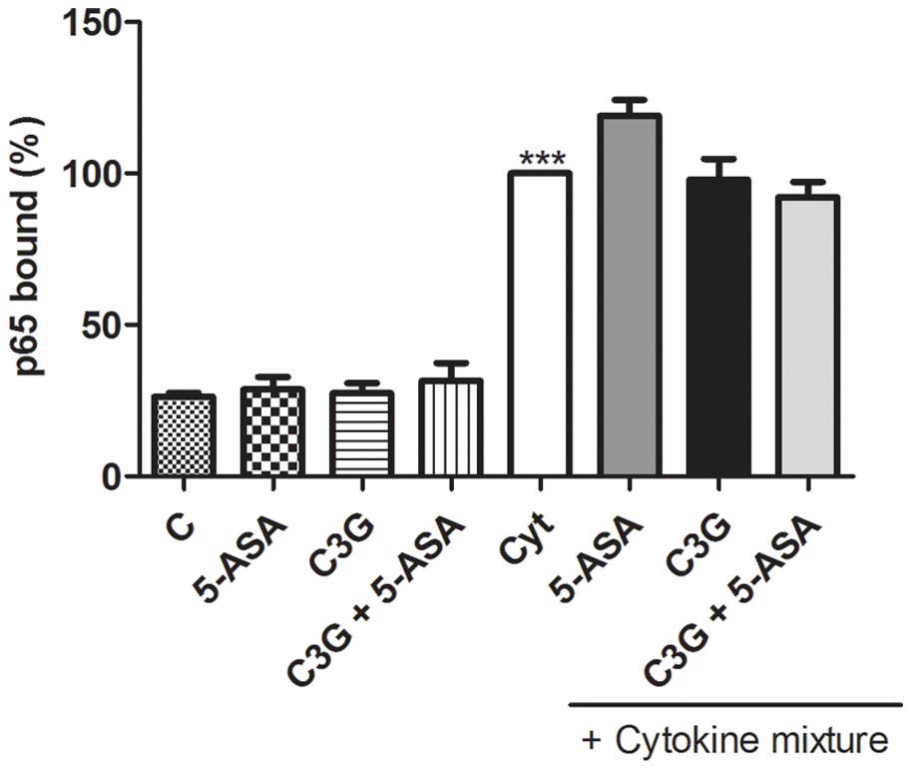
C3G and 5-ASA do not suppress the activation of NF-kB-p65 in HT-29 cells. Cells were pre-incubated with 25 µM C3G or 500 µM 5-ASA or both and then treated with a combination of cytokines for 30 minutes. NF-kB activation was evaluated in nuclear extracts by a DNA-binding activity assay. Values are mean ± SEM of at least three different experiments, in duplicate. ^***^ *P*<0.001 vs Control.

Given that p65 accumulation and DNA binding in the cell nucleus is critical in regulating the expression of target genes, we decided to determine whether the down-regulation of iNOS and COX-2 expression and the inhibition of pro-inflammatory mediators production, by the compounds under study, would be due to the suppression of NF-kB transcriptional activation. To test this hypothesis, cells were treated as referred and the DNA binding activity of p65 was measured, as described in materials and methods. As shown in Figure 6, although cells stimulation with cytokines increased the DNA binding activity of p65 up to approximately 4-fold, neither C3G nor 5-ASA alone or in combination interfered with p65 transcriptional activity, in our assay conditions.

### 5. C3G and/or 5-ASA reduced the levels of cytokine-induced phosphorylated STAT1 in the nucleus of HT-29 cells

**Figure 7:**
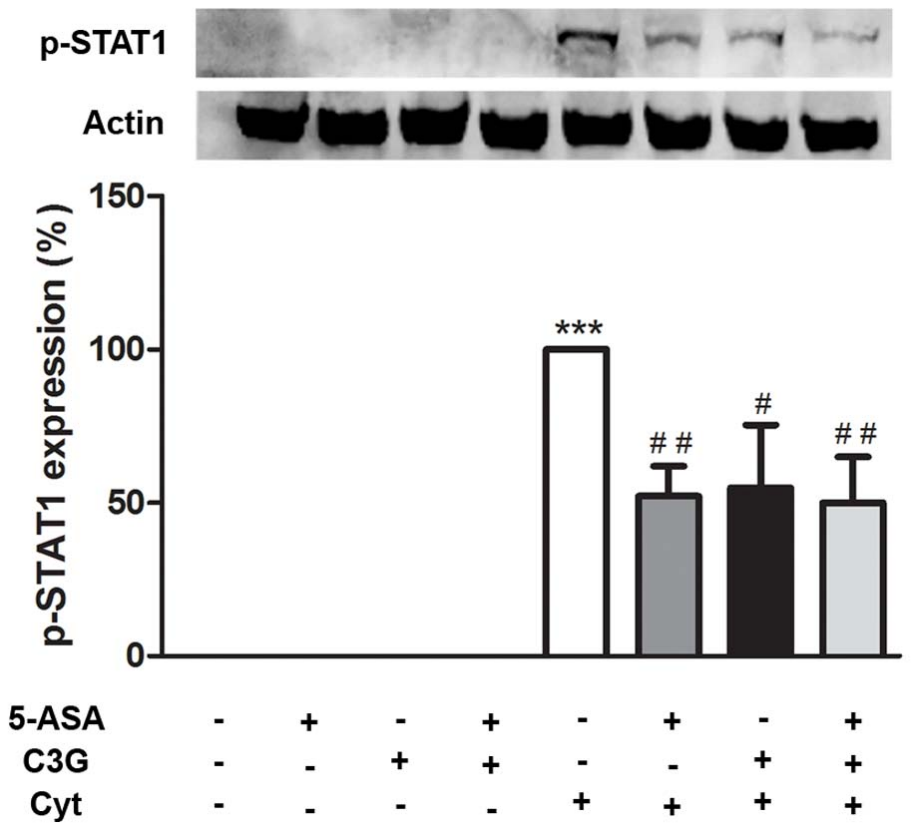
C3G and 5-ASA reduce the levels of cytokine-induced STAT1 activation in HT-29 cells. Cells were pre-incubated with 25 µM C3G or 500 µM 5-ASA or both and then treated with a combination of cytokines for 30 minutes. STAT1 phosphorylation was evaluated in nuclear extracts by Western blotting, as described in “Material and Methods”. Values are mean ± SEM of at least three different experiments, in duplicate. ^***^ *P*<0.001 vs Control, ^#^ *P*<0.05,^ ##^ *P*<0.01 vs Cytokines.

Considering that STAT1 is another important transcription factor that may be behind the protective effect of C3G and/or 5-ASA, we further examined the effect of these compounds on the levels of phosphorylated (activated) STAT1, in the cell nucleus. For this purpose, a Western-blotting analysis was carried out. As well as in the activation time course of NF-kB, cytokine-induced phosphorylation of STAT1 started at 15 minutes after the insult but became much stronger at 30 minutes (data not shown). It is worthwhile to note that, as illustrated in Figure 7, the pre-incubation with either C3G, 5-ASA or both led to a decrease in the nuclear content of this activated transcription factor, in about 50%. As happened with the other targets studied, the combination of C3G and 5-ASA did not show more benefit than the compounds alone.

### 6. C3G, like 5-ASA, did not affect the cytokine-induced phosphorylation of p38 MAPK, in HT-29 cells

**Figure 8:**
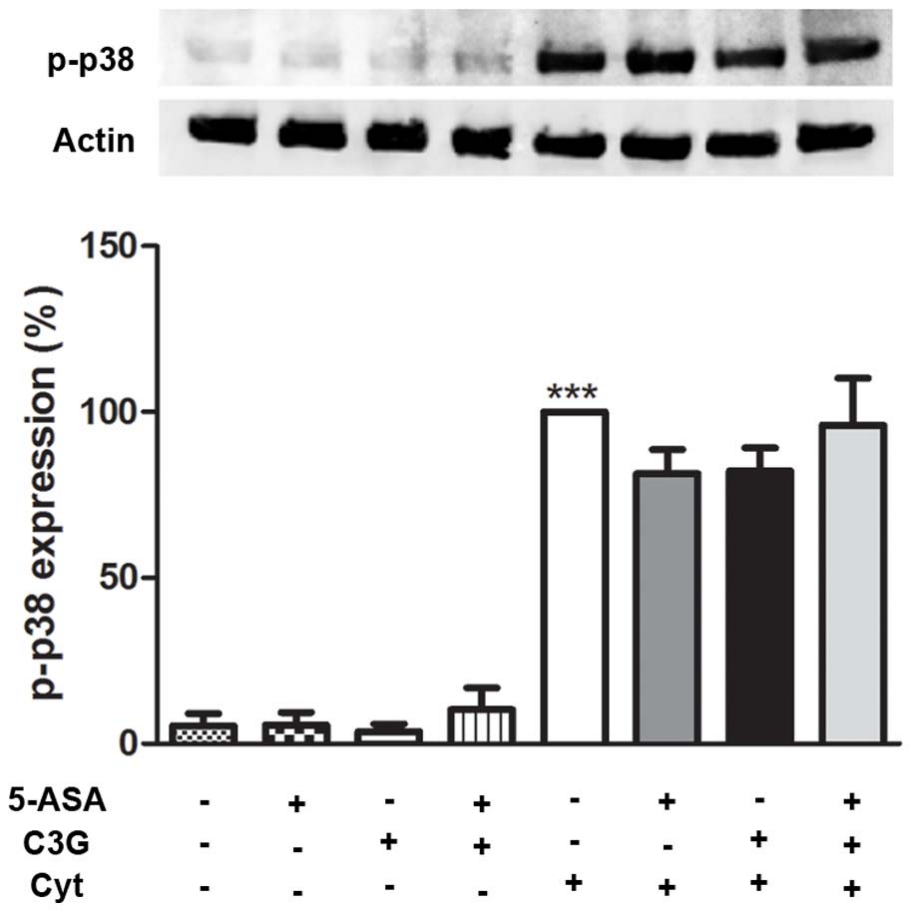
Failure of C3G and 5-ASA to inhibit cytokine-induced phosphorylation of p38 MAPK in HT-29 cells. Cells were pre-incubated with 25 µM C3G or 500 µM 5-ASA or both and then treated with a combination of cytokines for 30 minutes. Phosphorylation of p38 MAPK was evaluated in total extracts by Western blotting, as described in “Material and Methods”. Values are mean ± SEM of at least three different experiments, in duplicate. ^***^ *P*<0.001 vs Control.

Given that many transcription factors can be phosphorylated and activated by upstream kinases, like p38 MAPKs, it became important to explore whether anti-inflammatory action of C3G and 5-ASA was mediated through the p38 MAPK pathway in HT-29 cells. Thus, we next analyzed the ability of C3G and 5-ASA to inhibit the cytokine-induced phosphorylation of p38 MAPK. Treatment of cells with the mixture of cytokines significantly induced the phosphorylation of p38 MAPK at 30 minutes. However, at this time, C3G and/or 5-ASA did not counteract this process, as it is shown by Western-blotting technique (Figure 8).

## Discussion

In the last years, many studies have been carried out demonstrating the important role of anthocyanins in the prevention and treatment of chronic inflammatory diseases [5], [7]. It is known that health effects of polyphenols depend on the ingested amount and on the achieved bioavailability which deeply differs among the various polyphenols [8]. In what concerns anthocyanins, it is believed that their bioavailability is low, but it is also known that these compounds are unique because they can exist in many different molecular forms, in a dynamic equilibrium, and the currently used analytical methods underestimate the bioavailability data [2], [9]. Apart from this, many studies confirm that anthocyanins can be very active at the intestine level, reaching high concentrations in the gastrointestinal tract, which may be explained by their abundance in diet and poor absorption [8], [9]. This fact makes anthocyanins very attractive for exploring their anti-inflammatory potential in IBD context, in particular that of cyanidin-3-glucoside (C3G), one of the most abundant anthocyanins in nature.

The present study was undertaken to provide new insights into cellular signaling mechanisms underlying the ability of C3G to protect intestinal cells (HT-29), against pro-inflammatory stimulus, in comparison with 5-aminosalicylic acid (5-ASA). A combination of cytokines containing IL-1α, TNF-α, and IFN-γ was selected as a pro-inflammatory stimulus, since it is known that these cytokines are rapidly released by injured tissue or infection and are effective as inducers of the expression of different pro-inflammatory genes, depending on the cell type [24]–[26].

Thus, in the intestinal cell line used in this work, C3G was able to efficiently inhibit the NO production (Figure 3A) counteracting the iNOS expression (Figure 4A) like 5-ASA, but at a much lower concentration. NO is a free radical produced from the amino acid L-arginine by nitric oxide synthase (NOS) enzyme. Although NO, in constitutive levels, has a physiological role in maintaining adequate perfusion and regulation of microvascular and epithelial permeability [27], persistent overproduction of NO via up-regulation of iNOS is associated with inflammatory response leading to gut barrier injury [28]. The protective effects of C3G, with respect to iNOS and NO production, are very interesting because they revealed that this anthocyanin, which is a natural polyphenol widespread in plants, acted in a very similar way to 5-ASA, which in turn is a potent anti-inflammatory therapeutic agent, commonly used in clinical practice. Furthermore, C3G acted in a concentration 20 times lower than 5-ASA. Nonetheless, the combination of the two compounds did not provide better protection over the individual compounds, excluding both additive or synergistic effects.

In contrast to the effect on iNOS, C3G/5-ASA combination demonstrated a higher protection for COX-2 expression than that provided by the individual compounds (Figure 4B). COX-2 is the inducible form of COX and its expression is up-regulated in the inflamed gut of IBD patients [29]. Thus, high levels of prostaglandins have also been found in the mucosa of IBD patients [29] leading to the perpetuation of inflammation [30]. In this way, we also proved the ability of C3G to prevent PGE_2_ biosynthesis and in agreement with COX-2 results, C3G inhibited PGE_2_ production more significantly than 5-ASA (Figure 3B), bearing into consideration the much lower concentration of C3G used to achieve this inhibitory effect. However, the combined effects of C3G plus 5-ASA on PGE_2_ production were similar to that of C3G, revealing no advantage in adding 5-ASA over C3G. Also, of note is that this cell line expresses mRNA for IL-8 and secretes it after stimulation [31]. Actually, we observed such overproduction, but only C3G pre-treatment was able to significantly reduce it (Figure 3C).

Although it is known that the transcriptional regulation of iNOS and COX-2 is complex [32], [33], the nuclear factor kB is one of the most important regulators of pro-inflammatory genes expression and it is well-established that its activation is significantly induced in intestine of IBD patients [17], [34]. So, the effects observed in pre-treated HT-29 cells with either C3G or 5-ASA could be due to the suppression of NF-kB activation. This activation process can be initiated by a wide variety of different stimuli, which lead to the phosphorylation and degradation of the NF-kB inhibitory molecules, IkB proteins. [17]. In the present study, we observed that in our assay conditions the combination of cytokines was able to induce the degradation of IkB-α but, unexpectedly, neither C3G nor 5-ASA inhibited such degradation (Fig. 5). This degradative process is a crucial step for the activation of NF-kB with subsequent translocation to the nucleus and binding to DNA, in the classical pathway of NF-kB activation. However, recent evidences indicate that transcriptional activity of NF-kB also requires the direct modification of NF-kB proteins, namely by phosphorylation and acetylation. The loss of phosphorylation of p65 interferes with its DNA binding and transactivation activities [18], [35]–[37]. Taking this into account and considering that other authors have already reported the inhibitory effect of 5-ASA on inducible NF-kB-dependent transcription in intestinal epithelial cells, independent of preventing the IkB-α degradation [38], we investigated whether C3G or 5-ASA or both could hamper NF-kB activation, by interfering with its DNA binding. However, in our assay conditions, in cells pre-treated with the compounds no prevention of NF-kB activation was observed (Figure 6). Our findings seem to conflict with those of Min *et al* [39], who have reported an inhibitory effect of C3G on LPS-induced NF-kB activation in RAW 264.7 cells. These contradictory results might be explained by the differences in the cell type and in the pro-inflammatory stimulus.

Thus, our belief is that the anti-inflammatory effects of C3G observed in HT-29 cells, stimulated by a cocktail of cytokines, could be related to the suppression of an alternative cell signaling, other than NF-kB. This is consistent with reports by others showing that some polyphenols preferentially suppress STAT1 activation rather than NF-kB activation [40], [41]. The JAK-STAT signaling pathway is a common signaling pathway activated by various stimuli, namely interferons. The binding of a cytokine to its cell-surface receptor results in the activation of JAK tyrosine kinases, which in turn phosphorylate STATs. Then, STATs dimerize, translocate into the nucleus and activate the transcription of STAT-responsive genes, namely iNOS [42]–[45]. The present study demonstrated, for the first time, that pre-incubation of C3G in HT-29 cells decreased the nuclear levels of this activated transcription factor, to about 50% (Figure 7). A similar effect was obtained by 5-ASA but at a much higher concentration than that of C3G. One possible explanation to such decrease in nuclear activated STAT1 levels could be the induction of the expression of the SOCS family of proteins by our compounds. These proteins are in part in charge of the negative feedback mechanism engaged by STATs. They can block the recruitment of STATs, bind to JAKs, or even target STATs for proteossomal degradation [42], [43], [46]. On the other hand, another possible explanation will be that C3G and 5-ASA can induce the dephosphorylation of STAT1. Actually, it has been described that tyrosine-phosphorylated STAT1 requires to be dephosphorylated, by some nuclear phosphatases, in order to leave the nucleus [43], [47].

Furthermore, there are many kinase pathways involved in the regulation of inflammatory response upstream transcription factors, which may also be important to understand the mechanisms behind the anti-inflammatory effects of C3G, namely the p38 MAPK pathway. In fact, this pathway has already been identified as crucial for induction of iNOS and COX-2 in HT-29 cells by a mixture of cytokines [48]. However, under our experimental conditions none of the compounds was successful in inhibiting the phosphorylation (activation) of p38 MAPK, revealing that p38 MAPK was not involved in the protective effect of C3G and 5-ASA.

In conclusion, *in vitro*, under our experimental conditions, C3G showed to be effective in inhibiting cytokine-induced pro-inflammatory markers, namely NO, PGE_2,_ IL-8, iNOS and COX-2, without affecting the activation of either NF-kB or p38 MAPK but significantly decreasing the amount of activated STAT1 in the nucleus, in HT-29 cells. Moreover, we demonstrated, for the first time, that in comparison with 5-ASA, C3G has a stronger anti-inflammatory activity regarding the studied pro-inflammatory markers in particular, taking into account the difference in the concentrations used. However, it is known that, *in vivo,* anthocyanins suffer from spontaneous degradation and are metabolized by the indigenous microbiota population in the colon, which causes the release of aglycones of their glycosides and eventually the disruption of the ring [49], [50]. Thus, unanswered questions should lead to interesting future research to clarify the actual molecular structures underlying the protective effects of C3G and also to investigate the accessibility of them to the epithelium.

Despite this and considering that current treatment options in patients with IBD are not curative and patients face lifelong therapy, C3G may be envisaged as a promising nutraceutical giving complementary benefits in attenuating inflammation and decreasing the risk for the development of colorectal cancer observed in these patients.
